# Oncogenic KRAS blockade therapy: renewed enthusiasm and persistent challenges

**DOI:** 10.1186/s12943-021-01422-7

**Published:** 2021-10-04

**Authors:** Daolin Tang, Guido Kroemer, Rui Kang

**Affiliations:** 1 The Third Affiliated Hospital, Guangzhou Medical University, Guangzhou, China; 2 Department of Surgery, UT Southwestern Medical Center, Dallas, TX USA; 3 Centre de Recherche des Cordeliers, Equipe labellisée par la Ligue contre le cancer, Université de Paris, Sorbonne Université, INSERM U1138, Institut Universitaire de France, Paris, France; 4 Metabolomics and Cell Biology Platforms, Gustave Roussy Cancer Campus, Villejuif, France; 5 Pôle de Biologie, Hôpital Européen Georges Pompidou, AP-HP, Paris, France

**Keywords:** Gene mutation, Covalent inhibitor, Drug resistance, KRAS, Targeted therapy

## Abstract

Across a broad range of human cancers, gain-of-function mutations in *RAS* genes (*HRAS*, *NRAS*, and *KRAS*) lead to constitutive activity of oncoproteins responsible for tumorigenesis and cancer progression. The targeting of RAS with drugs is challenging because RAS lacks classic and tractable drug binding sites. Over the past 30 years, this perception has led to the pursuit of indirect routes for targeting RAS expression, processing, upstream regulators, or downstream effectors. After the discovery that the KRAS-G12C variant contains a druggable pocket below the switch-II loop region, it has become possible to design irreversible covalent inhibitors for the variant with improved potency, selectivity and bioavailability. Two such inhibitors, sotorasib (AMG 510) and adagrasib (MRTX849), were recently evaluated in phase I-III trials for the treatment of non-small cell lung cancer with KRAS-G12C mutations, heralding a new era of precision oncology. In this review, we outline the mutations and functions of KRAS in human tumors and then analyze indirect and direct approaches to shut down the oncogenic KRAS network. Specifically, we discuss the mechanistic principles, clinical features, and strategies for overcoming primary or secondary resistance to KRAS-G12C blockade.

## Key points

- *KRAS* is the most frequently mutated oncogene in human cancer and has challenged the development of clinical anticancer therapeutics in the last 30 years.
- Mutated KRAS oncoprotein disrupts GAP-mediated GTP hydrolysis and thus remains in a continuous GTP binding activation state.
- Small-molecule inhibitors that directly target KRAS-G12C mutants provide new tools for precision oncology.
- Clinical trials involving covalent KRAS-G12C inhibitors (adagrasib and sotorasib) have shown promising activity against lung cancers harboring KRAS-G12C mutations.
- Secondary KRAS mutations, gain-of-function mutations of the MAPK pathway, loss-of-function mutations in tumor suppressor genes, and other gene alterations are conducive to acquired resistance to KRAS-G12C inhibitors.
- The design and implementation of strategies to minimize or overcome drug resistance is an important goal for the further development of KRAS inhibitors.

## Introduction

The *RAS* gene was initially identified as a virus-encoded gene in 1964 [1], and later was found to be a genome-encoded oncogene that is frequently mutated in human cancers [2]. Thus, activating mutations of *RAS* are found in 19% of neoplasias, corresponding to approximately 3.4 million new diagnoses of malignant disease worldwide each year [2]. The *RAS* gene family includes *HRAS*, *NRAS*, and *KRAS*, which are encoding proteins that play partially overlapping but specific roles in signaling transduction [3]. For example, the knockout of *Kras* is embryonically lethal in mice, while the depletion of *Nras* or *Hras* does not affect development [4]. The mutation of human *KRAS* was first detected in non-small cell lung cancer (NSCLC) [5–7]. The earliest evidence that *RAS* is an oncogene was based on the fact that transfecting mouse *Kras* causes morphological transformation of NIH-3T3 fibroblasts [8]. Subsequent studies involving transgenic mice confirmed that the mutation of *Hras*, *Nras*, or *Kras* mimicked human oncogenesis by triggering the stochastic transformation of cells [9–12]. Clinical studies revealed the prognostic impact of *RAS* mutations in certain cancers [2]. However, in spite of decades-long efforts of academia and industry to target RAS protein for cancer therapy [13], the design of direct RAS pharmacological inhibitors has only achieved a major breakthrough during recent years [14]. In 2021, two covalent inhibitors of KRAS-G12C protein (hereafter referred to as G12Ci), sotorasib (AMG 510) [15] and adagrasib (MRTX849) [16], were clinically approved by the U.S. Food and Drug Administration and the European Medicines Agency to treat patients with advanced NSCLC carrying the *KRAS-G12C* mutation. Monotherapies or combination therapies using G12Ci are being evaluated in clinical trials for advanced or metastatic solid cancer, including NSCLC, colorectal cancer (CRC), and pancreatic ductal adenocarcinoma (PDAC) [17]. This progress is inspiring scientists to continue to design drugs targeting key oncogenic drivers, even those that previously were considered to be “undruggable” like KRAS [18].

Here, we summarize recent therapeutic advances in mutant KRAS blockade that have led to clinical approval or are currently being evaluated in trials (Table 1), while discussing attempts to target upstream regulators, downstream effectors, and mutant KRAS protein itself. We also discuss the challenges associated with G12Ci-based treatments and the future prospects of this evolving topic.

**Table 1:** Clinical trials targeting KRAS

Target	Agent	Combinations	Study phase	Tumor type	Recruitment status	Trial number
BRAF, CRAF	LXH-254	None	I	Advanced solid tumors harboring MAPK pathway alterations	Active, not recruiting	NCT02607813
BRAF, CRAF	LXH-254	Rineterkib (RAF/ERK inhibitor); trametinib (MEK inhibitor); ribociclib (CDK4/6 inhibitor); EGF816 (EGFR inhibitor); dabrafenib (BRAF mutant inhibitor)	I, II	Unresectable or metastatic melanoma; EGFR-mutant NSCLC	Recruiting; active, not recruiting	NCT04417621; NCT02974725; NCT03333343; NCT04294160
ERK	LY3214996; GDC-0994; ulixertinib; MK-8353	None	I, II	Acute myeloid leukemia; locally advanced or metastatic solid tumors; metastatic uveal melanoma; acute myelogenous leukemia or myelodysplastic syndromes	Recruiting; completed; terminated	NCT04081259; NCT01875705; NCT04488003; NCT03417739; NCT02296242; NCT01358331
ERK	LY3214996	RMC-4630 (SHP2-inhibitor); abemaciclib (CDK4/6 inhibitor); hydroxychloroquine (autophagy inhibitor)	I, II	Metastatic KRAS mutant cancers; solid tumors harboring pathogenic alterations in BRAF, RAF1, MEK1/2, ERK1/2, and NF1	Not yet recruiting	NCT04916236; NCT04956640; NCT04534283; NCT04616183; NCT04391595; NCT04386057
ERK	GDC-0994	Cobimetinib (MEK inhibitor)	I	Locally advanced or metastatic solid tumors	Completed	NCT02457793
ERK	Ulixertinib	Hydroxychloroquine (autophagy inhibitor); palbociclib (CDK4/6 inhibitor)	I, II	Advanced MAPK-mutated gastrointestinal adenocarcinomas; advanced pancreatic and other solid tumors	Recruiting	NCT041452973; NCT03454035
ERK	MK-8353	Selumetinib (MEK inhibitor); pembrolizumab (anti–PD-1 ab)	I	Advanced malignancies	Completed; active, not recruiting	NCT03745989; NCT02972034
KRAS	AZD4785	None	I	Advanced solid tumors	Completed	NCT03101839
KRAS-G12C	Sotorasib	None	I, II	KRAS-G12C–mutant advanced/metastatic solid tumors	Recruiting; not yet recruiting	NCT04380753; NCT04625647; NCT04667234; NCT04933695
KRAS-G12C	Sotorasib	Docetaxel (microtubule inhibitor); pembrolizumab (anti–PD-1 ab)	I, II, III	KRAS-G12C–mutant advanced/metastatic solid tumors	Active, not recruiting; recruiting	NCT04303780; NCT03600883; NCT04613596
KRAS-G12C	Adagrasib	Docetaxel (microtubule inhibitor); pembrolizumab (anti–PD-1 ab); cetuximab (anti-EGFR ab); afatinib (pan-EGFR inhibitor); TNO155 (SHP2 inhibitor)	I, II, III	KRAS-G12C–mutant advanced/metastatic solid tumors	Recruiting	NCT04685135; NCT03785249; NCT04330664; NCT04793958
KRAS-G12C	GDC-6036	Atezolizumab (anti–PD-L1 ab); cetuximab (anti-EGFR ab); bevacizumab (anti-VEGF ab); erlotinib (EGFR inhibitor)	I	KRAS-G12C–mutant advanced/metastatic solid tumors	Recruiting	NCT04449874
KRAS-G12C	D-1553	Standard treatment	I	KRAS-G12C–mutant advanced/metastatic solid tumors	Recruiting	NCT04585035
KRAS-G12C	JNJ-74699157	Standard treatment	I	KRAS-G12C–mutant advanced/metastatic solid tumors	Completed	NCT04006301
KRAS-G12C	LY3499446	Abemaciclib (CDK4/6 inhibitor); cetuximab (anti-EGFR ab); erlotinib (EGFR inhibitor); docetaxel (microtubule inhibitor)	I, II	KRAS-G12C–mutant advanced/metastatic solid tumors	Terminated	NCT04165031
KRAS-G12D	siG12D-LODER	Gemcitabine + nab-paclitaxel; FOLFIRINOX	II	Advanced pancreatic cancer	Recruiting	NCT01676259
MEK	Cobimetinib	Belvarafenib (RAF inhibitor)	I	Advanced or metastatic solid tumors	Recruiting	NCT03284502
MEK	Trametinib	LXH254 (RAF inhibitor)	I	NSCLC or melanoma	Recruiting	NCT02974725
MEK	Pimasertib	None	I	N-RAS–mutated locally advanced or metastasis malignant cutaneous melanoma	Recruiting	NCT01693068, NCT00982865
MEK	Pimasertib	SAR405838 (MDM2 antagonist)	I	Advanced solid tumors	Completed	NCT01985191
MEK	Mirdametinib	Lifirfenib	I	Advanced or refractory solid tumors	Recruiting	NCT03905148
p110α	Alpelisib	Capecitabine (nucleoside metabolic inhibitor); paclitaxel (microtubule inhibitor)	I	Patients with PIK3CA mutant metastatic colorectal cancer; PIK3CA-altered metastatic/recurrent gastric cancer	Not yet recruiting	NCT04753203; NCT04526470
p110α	GDC-0077	Entrectinib (pan-TRK inhibitor)	I	Breast cancer and advanced solid tumors	Recruiting	NCT04632992
RAF	PLX8394; TAK-580	None	I, II	Advanced unresectable solid tumors; low-grade glioma	Recruiting	NCT02428712; NCT03429803
RAF	Belvarafenib	None	I	Solid tumors	Completed	NCT02405065
RAF	Belvarafenib	Cobimetinib (MEK inhibitor); cetuximab (anti-EGFR ab); atezolizumab (anti–PD-L1 ab)	I	Advanced or metastatic solid tumors; NRAS-mutant advanced melanoma	Recruiting	NCT03284502; NCT04835805
RAF, EGFR	Lifirfenib	None	I	Solid tumors	Completed	NCT02610361; NCT03641586
RAF, EGFR	Lifirfenib	Mirdametinib (MEK inhibitor)	I	NSCLC with confirmed KRAS mutations	Recruiting	NCT04294160
SHP2	BBP-398; JAB-3068; RMC-4630; RLY-1971; JAB-3312; SH3809	None	I, II	Advanced or metastatic solid tumors	Recruiting	NCT04528836; NCT03565003; NCT03518554; NCT03634982; NCT04252339; NCT04121286; NCT04045496; NCT04843033
SHP2	RMC-4630	LY3214996 (ERK inhibitor); cobimetinib (MEK inhibitor); osimertinib (EGFR inhibitor)	I, II	Advanced or metastatic solid tumors	Not yet recruiting; recruiting	NCT04916236; NCT03989115
SHP2	ERAS-601	Cobimetinib (MEK inhibitor)	I	Advanced or metastatic solid tumors	Recruiting	NCT04670679
SHP2	TNO155	Nazartinib (EGFR inhibitor); spartalizumab (anti–IL-1β antibody); ribociclib (CDK4/6 inhibitor); adagrasib (KRAS-G12C inhibitor); JDQ443 (KRAS-G12C inhibitor)	I, II	Advanced solid tumors	Recruiting	NCT03114319; NCT04000529; NCT04330664; NCT04699188
SOS1	BI 1701963	Trametinib (MEK inhibitor); BI 3011441 (MEK inhibitor); irinotecan (topoisomerase I inhibitor)	I	Advanced or metastatic solid tumors	Recruiting	NCT04111458; NCT04835714; NCT04627142

### Type and frequency of *KRAS* mutation

**Fig. 1:**
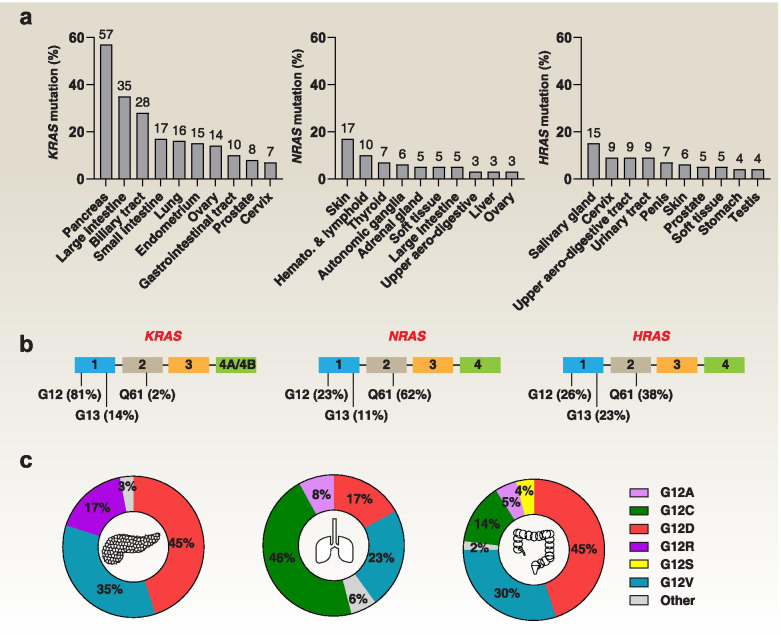
Type and frequency of RAS mutations in human cancers. **a.** Somatic mutations of RAS oncogene in the top 10 human cancers. **b.** The frequency and location of G12, G13, and Q61 mutations in the exons of RAS oncogenes. **c.** The frequency and type of KRAS mutations in codon 12 in pancreatic cancer, colorectal cancer, and lung adenocarcinoma. The data were derived from recent studies using the COSMIC or cBioPortal database [2, 14, 19]

*RAS* genes are mutated at different prevalence rates in human cancers (Fig. 1a) [19]. Elucidating similarities and differences among these *RAS* mutations from a developmental or evolutionary perspective remains a challenge [20, 21]. Some *RAS* gene mutations are innocuous, but others cause cancer by producing oncoproteins. For example, three amino acid residues (G12, G13, and Q61) in HRAS, KRAS, and NRAS are mutational hot spots, though with distinct frequencies in different human tumor types (Fig. 1b) [14]. A *KRAS-G12* mutation is a common event in pancreatic (91%), colorectal (68%), and lung adenocarcinoma (85%; a subtype of NSCLC) [14]. Among these, *KRAS-G12D* is the leading mutation in pancreatic (45%) and colorectal adenocarcinoma (45%), while *KRAS-G12C* mainly occurs in lung adenocarcinoma (46%) (Fig. 1c) [14]. In contrast, 85% of human melanomas have *KRAS-Q61* mutations, especially *Q61R* (46%) [14]. Whole-exome sequencing of human PDAC or NSCLC tumors shows that, despite the genetic heterogeneity within the tumor, *KRAS* is mutated in different regions [22, 23]. Consistent with this, the co-mutational interactions between each *KRAS* allele and other unrelated genes are highly tissue-specific, emphasizing the complexity of cell type-specific oncogenesis [24]. *KRAS* mutations usually co-occur with mutations in tumor protein p53 (*TP53*) and cyclin-dependent kinase inhibitor 2A (*CDKN2A*) in PDAC, Kelch-like ECH-associated protein 1 (*KEAP1*) and/or serine/threonine kinase 11 (*STK11*) in NSCLC, or APC regulator of WNT signaling pathway (*APC*) and phosphatidylinositol-4,5-bisphosphate 3-kinase catalytic subunit alpha (*PIK3CA*) in CRC. While mutations in exon 2 of *KRAS* are the most common, it is important to note that they affect the differential use of exon 4, giving rise to two splice variants, KRAS4A and KRAS4B (Fig. 1b) [25]. These KRAS variants differ in their C-terminal membrane targeting region, posttranslational modifications, and interactomes, thus exhibiting different signal behaviors in development, metabolism, and proliferation [26–28]. Therefore, to target KRAS, investigators must consider changes in the protein structure caused by point mutations, but also isoform-specific properties [29].

### Prognostic and predictive value of *KRAS* mutations

Depending on the clinical settings, the prognostic and predictive values of *KRAS* mutations are variable and even conflicting. For example, an early study of lung adenocarcinoma patients showed that the *KRAS* mutation in code 12 is an unfavorable prognostic factor [30]. Similarly, a meta-analysis found that *KRAS* mutations are associated with poor survival in patients with early resectable NSCLC [31]. In contrast, a pooled analysis of NSCLC patients treated with cisplatin-based chemotherapy revealed that a KRAS mutation was not a prognostic factor [32]. Of note, a *KRAS* mutation is a negative predictor of response to epidermal growth factor receptor (EGFR) tyrosine kinase inhibitors (TKIs; e.g., cetuximab, gefitinib, or erlotinib) (Table 2) [33–36], but a positive predictor of response to immune checkpoint inhibitors (ICIs; including anti–PD-1 and anti–PD-L1) (Table 3) [37–39]. However, response rates are variable and dissenting reports suggest that *KRAS* mutations cannot guide the therapeutic choice between TKIs and ICIs in NSCLC patients [40, 41].

**Table 2:** Tyrosine kinase inhibitors

Tyrosine kinase inhibitors (TKIs) are a group of drugs that disrupt the tyrosine kinase (TK) signal transduction pathway through a variety of mechanisms. They can compete with adenosine triphosphate (ATP), phosphorylated entities, substrates, or can act in an allosteric manner, that is, bind to sites outside the active site and affect the sites’ activity through conformational changes. TKs can be divided into receptor tyrosine kinases (RTKs), nonreceptor tyrosine kinases (NRTKs), and dual-specific kinases (DSKs). DSKs phosphorylate serine, threonine, and tyrosine residues. Approximately 20 different transmembrane RTK subfamilies have been identified, such the families for vascular endothelial growth factor receptor (VEGFR), platelet-derived growth factor receptor (PDGFR), insulin receptor (INSR), fibroblast growth factor receptor (FGFR), and epidermal growth factor receptor (EGFR). NRTKs are cytoplasmic proteins and do not have a transmembrane domain. NRTKs are mainly composed of nine families, including those for Abl, Ack, Csk, Fak, Fes/Fer, Jak, Src, Syk/Zap70, and Tec. The most typical example of DSK is mitogen-activated protein kinase kinase (MEK), which is involved in the mitogen-activated protein kinase (MAPK) pathway. More than 50 FDA-approved TKIs (including small-molecule inhibitors and monoclonal antibodies) are used to treat various diseases, including cancer.

**Table 3:** Immune checkpoint inhibitors

Immune checkpoint inhibitors (ICIs) are a group of drugs that inhibit the activity and function of inhibitory immune checkpoint molecules, such as programmed cell death protein 1 (PD-1), programmed death ligand 1 (PD-L1), cytotoxic T lymphocyte-associated protein 4 (CTLA-4), lymphocyte activation gene 3 (LAG3), and T-cell immunoglobulin and mucin domain-containing protein 3 (TIM3). Under physiological conditions, inhibitory immune checkpoint molecules play an important role in maintaining self-tolerance, preventing autoimmune reactions, and minimizing tissue damage by regulating the duration and intensity of immune responses. However, abnormal expression and excessive activation of immune checkpoint molecules can cause many diseases, including cancer. In particular, inhibitory immune checkpoint molecules are upregulated in various cells within the tumor microenvironment, forming various pairings and limiting the normal antitumor function of immune cells. In contrast, the use of ICIs can restore the function of immune cells hijacked by cancer cells, resulting in an enhanced immunosurveillance with a cytotoxic T lymphocyte (CTL) response. ICIs (e.g., pembrolizumab, nivolumab, cemiplimab, and atezolizumab) have changed the landscape of cancer treatment and become a new hope for cancer patients after the failure of regular chemotherapy or radiotherapy.	

In PDAC, patients with *KRAS-G12D* mutations (but not total *KRAS* mutations) have a worse prognosis than patients with wild-type *KRAS* [42–44]. Other studies suggest that the prognosis of *KRAS-G12V* is poorer than that for other mutations [45, 46], commensurate to a *KRAS-G12V–*associated increase in circulating regulatory T cells that most likely limits antitumor immunity [47]. In advanced PDAC, *KRAS* mutation status is predictive for the efficacy of erlotinib rather than prognostic [48]. This contradicts other studies reporting that *KRAS* wild-type patients with PDAC have a significant advantage after treatment with gemcitabine/nimotuzumab [49] or gemcitabine/erlotinib [50] with respect to overall survival. Circulating tumor DNA (ctDNA) has recently become a minimally invasive tool used in precision oncology to evaluate genetic alterations. Mutant *KRAS* in ctDNA might be a more sensitive predictor of survival than the ELISA-based detection of cancer antigen 19-9 (CA 19-9) [51, 52]. Before a clear conclusion can be drawn regarding the impact of *KRAS* mutations on overall survival in PDAC [53, 54], additional data from analyses of other genetic alterations are needed.

In localized CRC, *KRAS* mutations usually suggest a poor prognosis [55–57]. Some *KRAS* mutations, including *KRAS-G12V*, are more aggressive than others [58]. In contrast to *BRAF* mutations, *KRAS* mutations have no major prognostic value in advanced CRC patients [59]. The association between *KRAS* mutations and poor clinical outcomes from TKI treatment has been confirmed in several independent studies [60–63]. Compared with *KRAS-G12V* mutations or wild-type tumor groups, CRC patients with *KRAS-G13D* mutations are insensitive to cetuximab therapy [64, 65]. Nevertheless, the impact of *KRAS* mutations on cetuximab treatment needs to be further evaluated in prospective randomized trials. Reportedly, *KRAS-G12D–*mediated inhibition of interferon regulatory factor 2 (IRF2) drives CRC resistance to anti–PD-1 therapy in preclinical models [66]. However, the clinical implications of these findings remain elusive.

In conclusion, the prognostic and predictive values of *KRAS* mutations are affected by many factors, such as tumor type, stage, patient age, sex, the coexistence of mutations affecting other oncogenes or tumor suppressors, and treatment regimens. For this reason, the clinical utility of detecting *KRAS* mutations may be over- or underestimated. Careful meta-analyses that avoid nonrandom systematic errors due to variations between trials [67] are needed to clarify this issue.

### Activation and modulation of *KRAS* mutations

**Fig. 2:**
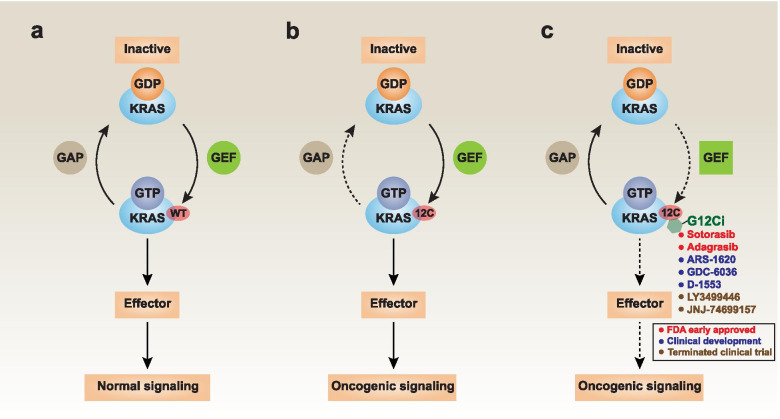
Principle of inhibiting oncogenic KRAS activation. **a.** The wild-type (WT) KRAS protein maintains a balance between the inactive state of guanosine diphosphate (GDP) binding and the active state of guanosine triphosphate (GTP) binding. This process is mediated by GTPase activating protein (GAP) and guanine nucleotide exchange factor (GEF). **b.** The KRAS oncoprotein (e.g., KRAS-G12C) disrupts GAP-mediated GTP hydrolysis, allowing these mutants to accumulate in a continuous GTP-binding active state, which is responsible for oncogenic activity. **c.** The covalent inhibitor of KRAS-G12C protein (G12Ci) achieves allosteric inhibition of mutant cysteine 12 (12C) to prevent GEF-catalyzed nucleotide exchange and block subsequent effector pathways

Wild-type KRAS protein mostly resides on the cytoplasmic side of the plasma membrane, as well as at membranes of intracellular organelles, and is guided by protein localization signals (such as lipid moieties added to the carboxyl terminus) [68]. The RAS family of proteins belongs to a class of enzymes called small GTPases, which play a central role in cell signal transduction [69]. The functions and activities of RAS protein depend on the transition from an inactive guanosine diphosphate (GDP)-bound state to an active guanosine triphosphate (GTP)-bound state (Fig. 2a) [69]. Normally, this conversion process of RAS status is reversible and is maintained in a balanced manner by GTPase activating proteins (GAPs) and guanine nucleotide exchange factors (GEFs). GAPs, such as neurofibromin 1 (NF1), accelerate the GTP hydrolysis of RAS, leading RAS to an inactive state [70, 71]. GEFs, including the most universally expressed SOS Ras/Rac guanine nucleotide exchange factor 1 (SOS1), are responsible for producing active GTP-bound RAS [72–74]. The balance between hydrolysis and exchange determines the level of activated KRAS in the cell [75]. Oncogenic mutations of RAS disrupt GAP-mediated GTP hydrolysis, allowing these oncoproteins to accumulate in a continuous GTP-binding active state (Fig. 2b) [13]. Among distinct KRAS mutants, the KRAS-G12C protein exhibits the highest intrinsic GTP hydrolysis rate [76].

**Fig. 3:**
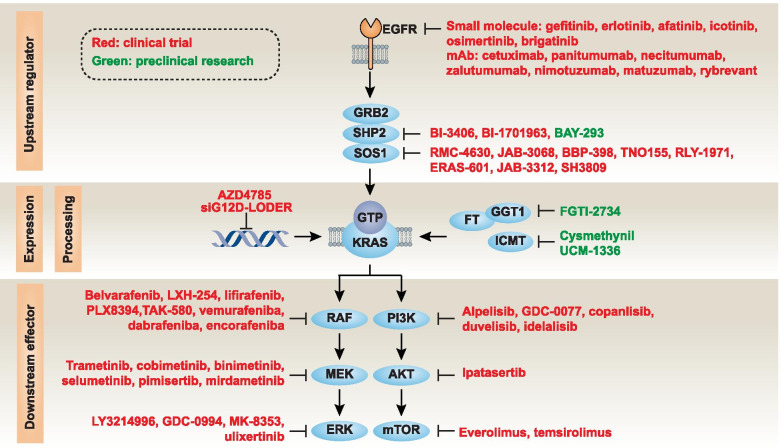
Indirect KRAS suppression strategy. The activation of receptor tyrosine kinases, such as members of the epidermal growth factor receptor (EGFR) family, activate KRAS through the growth factor receptor-bound protein 2 (GRB2)-SH2–containing protein tyrosine phosphatase 2 (SHP2)-SOS Ras/Rac guanine nucleotide exchange factor 1 (SOS1) pathway. The mutant KRAS protein accumulates in the guanosine triphosphate (GTP)-bound state, leading to the activation of downstream effector pathways, especially the RAF-MEK-extracellular signal regulated kinase (ERK) and the phosphatidylinositol 3-kinase (PI3K)-AKT-mechanistic target of rapamycin (mTOR) pathways. The localization of KRAS on the cell membrane is the first step in subsequent KRAS activation, which is mediated by enzymes, including but not limited to farnesyltransferase (FT), geranylgeranyltransferase 1 (GGT1), and isoprenylcysteine carboxyl methyltransferase (ICMT). In addition to directly inhibiting KRAS (exemplified by covalent allele-specific inhibitors that bind to KRAS-G12C), multiple approaches can indirectly inhibit the oncogenic pathway of KRAS by targeting upstream regulators, downstream effectors, and KRAS expression and processing. The main drugs or reagents used for indirect KRAS inhibition are shown in red (for clinical trials or approved for use in patients) or green (for preclinical research)

The upstream regulators of the RAS pathway involve receptor tyrosine kinases (RTKs), which are cell surface receptors for many growth factors, cytokines, and hormones (Fig. 3). One particular RTK subclass, the EGFR family, is composed of four closely related members, EGFR (also known as ERBB1 or HER1), ERBB2/HER2, ERBB3/HER3, and ERBB4/HER4. EGFR/ERBB1, the best characterized activator of RAS signaling, acts through binding to an adaptor protein, namely growth factor receptor bound protein 2 (GRB2) [77, 78]. GRB2 further mediates the recruitment and activation of SOS1- and SH2-containing protein tyrosine phosphatase 2 (SHP2) to activate GTP-bound RAS [79–84]. Activated EGFR mutations are often found in human cancers, especially NSCLC, and lead to the constitutive activation of downstream signals, including RAS [85]. G-protein–coupled receptors (GPCRs), the largest group of membrane receptors, also participate in RAS activation [86, 87]. Thus, the interplay between RTKs and GPCRs may increase the plasticity of RAS activation.

**Fig. 4:**
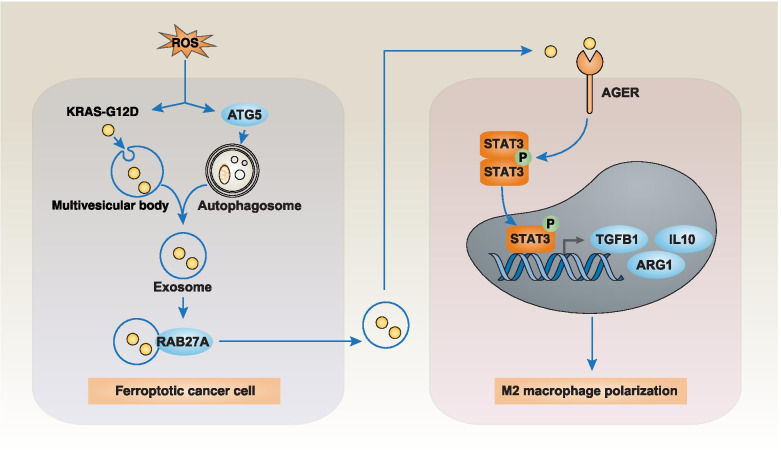
The immunosuppressive function of extracellular KRAS-G12D protein in the tumor microenvironment. KRAS-G12D protein can be released during ferroptosis, which is a regulated cell death caused by reactive oxygen species (ROS) and subsequent lipid peroxidation. The release of KRAS-G12D protein is mediated by exosomes, which are cargo extracellular vesicles produced by multivesicular bodies derived from endosomes. The small GTPase RAB27A regulates exocytosis of multivesicular endosomes, which leads to exosome secretion. This process is further enhanced by autophagy-related 5 (ATG5)-dependent autophagosome formation and autophagy-meditated secretion. Once released, the extracellular KRAS-G12D protein from exosomes is taken up by advanced glycosylation end product-specific receptor (AGER) on macrophages, leading to phosphorylation and activation of signal transducer and activator of transcription 3 (STAT3). Nuclear STAT3 acts as a transcription factor to produce cytokines, such as transforming growth factor beta 1 (TGFB1), interleukin 10 (IL10), and arginase 1 (ARG1), for polarization of M2 macrophages, which limits antitumor immunity

The downstream effectors of the RAS pathway are mainly involved in the activation of the mitogen-activated protein kinase (MAPK) and phosphatidylinositol 3-kinase (PI3K) pathways, which favor anabolic processes including cell growth, protein translation, and proliferation (Fig. 3) [88, 89]. Thus, the constitutive activation of membrane RAS-dependent signal pathways favors oncogenesis [90]. The aggregation of mitochondrial KRAS-G12V protein also favors tumor cell growth through metabolic effects [91]. Another distinctive feature of KRAS-G12D protein is that it can be released by PDAC cells into the tumor microenvironment and then mediates the polarization of pro-tumor macrophages (Fig. 4) [92]. Indeed, PDAC patients with KRAS-G12D–positive macrophages exhibit low survival rates [92]. There is also strong preclinical evidence that mutated *KRAS* requires additional factor (such as chronic inflammation or a high-fat or high-iron diet) to deploy its full carcinogenic activity [93–95]. Further work is needed to elucidate the likely complex cell-autonomous and non-autonomous effects of mutated and unmutated KRAS protein on the tumor microenvironment [21].

### Indirect KRAS suppression strategies

The most successful way to inhibit oncogenic kinases is to develop inhibitors that compete with ATP to bind to the kinase domain. However, KRAS uses GTP instead of ATP as a phosphate donor for signal transmission. Attempts to directly enzymatically inhibit KRAS function have been largely frustrated, leading to the development of indirect methods for KRAS inhibition. Below, we highlight some representative drugs that exemplify the main strategies for indirectly targeting mutant KRAS (Table 1 and Fig. 3).

#### Inhibition of KRAS expression

AZD4785 is a constrained ethyl-containing antisense oligonucleotide that is complementary to the sequence of the 3'UTR of *KRAS* mRNA, leading to the downregulation of wild-type and mutant KRAS protein [96]. AZD4785 selectively inhibits the proliferation of mutant *KRAS*-driven tumor cells *in vitro* or in xenograft models [96, 97]. However, intravenously infused AZD4785 failed to completely reduce *KRAS* mRNA in patients with NSCLC (NCT03101839) [14], calling for adjustments of the dose and method of administration. Another approach for transcriptionally inhibiting KRAS expression involves the use of a specific small interfering RNA (siRNA) named siG12D-LODER that specifically targets G12D but not wild-type KRAS. This agent showed promise in a phase I study (NCT01188785) in combination with chemotherapy (gemcitabine or FOLFIRINOX, i.e., a combination of 5-fluorouracil, leucovorin, irinotecan, and oxaliplatin) in 12 patients with advanced PDAC [98]. A phase II study (NCT01676259) evaluating this therapeutic strategy is now underway.

#### Inhibition of KRAS processing

The location of RAS on the cell membrane is the initial step of RAS activation and requires multiple posttranslational processing steps, especially lipid-related prenylation [99]. The two enzymes involved in KRAS prenylation are farnesyltransferase (FT) and geranylgeranyltransferase 1 (GGT1). Although preclinical studies suggest anticancer activity for FT inhibitors (for example, tipifarnib/R115777 and lonafarnib/SCH 66336) against *RAS*-mutant tumors, clinical studies have been disappointing [100–103]. One possible explanation for this failure is functional redundancy among FT and GGT1 [104]. A single molecule with dual inhibitory activity on FT and GGT1, such as FGTI-2734 [105], might have the potential to eliminate *RAS*-mutant tumors. Alternatively, targeting downstream RAS processing enzymes, such as isoprenylcysteine carboxyl methyltransferase (ICMT), may be attempted. Two ICMT inhibitors (cysmethynil and UCM-1336) impair the membrane localization of RAS (including that of KRAS) [106], but their application *in vivo* remains to be studied. Once RAS is effectively processed, membrane RAS protein undergoes activating self-association, and this process can be blocked by a synthetic binding protein called NS1 [107]. Since NS1 is an alien protein, its possible recognition by the immune system needs to be evaluated before it is introduced into clinical trials.

#### Inhibition of upstream signaling molecules

Gefitinib, erlotinib, afatinib (pan-EGFR inhibitors), icotinib, and osimertinib are first-line EGFR TKIs for treating NSCLC patients with *EGFR* mutations [108]. Lapatinib is the first dual inhibitor of EGFR and ERBB2/HER2 for treating *ERBB2*-positive breast cancer, whereas brigatinib is a mixed inhibitor of ALK and EGFR used for the treatment of metastatic NSCLC. The clinical benefit of small-molecule EGFR inhibitors on *KRAS*-mutant cancers is context-dependent. For example, gefitinib alone is not effective against *KRAS*-mutant NSCLC [109], while the combination of erlotinib and gemcitabine provides transient benefit to patients with *KRAS*-mutant PDAC [110]. Another approach to inhibit EGFR activity consists of the use of monoclonal antibodies [108]. Cetuximab and panitumumab are approved for metastatic CRC, while necitumumab is used for the treatment of squamous NSCLC. However, some studies suggest that antibodies against EGFR have no effect on *KRAS*-mutant CRC [60, 62], while others report that CRC patients with *KRAS-G13D* are sensitive to cetuximab treatment [65]. Regardless, acquired *KRAS* mutations are a common mechanism of resistance to EGFR inhibitors [111]. Recently, rybrevant, a bispecific antibody against EGFR and MET receptors, has been approved for the treatment of NSCLC patients with EGFR exon 20 insertion mutations. Other EGFR-specific monoclonal antibodies in clinical development are zalutumumab, nimotuzumab, and matuzumab. It will be important to understand how preexisting or acquired *KRAS* mutations will affect the clinical activity of such drugs.

The proteins SOS1 and SOS2 promote RAS activation by binding to GRB2. The SOS1 inhibitor BI-1701963 is used in combination with MEK inhibitors (trametinib and BI-3011441) or a topoisomerase I inhibitor (irinotecan) in clinical trials enrolling patients with advanced or metastatic solid cancer (Table 1). BI-1701963 has been designed to bind to the catalytic domain of SOS1 to prevent its interaction with KRAS-GDP [79]. SOS1 mutations lead to dysregulated enzymatic activities, which may cause drug resistance [112]. Because there is currently no SOS2-specific inhibitor, it is unclear whether targeting SOS2 would have the same effects as SOS1 inhibitors. It can be speculated that pan-SOS inhibitors might be particularly efficient in blocking the activation of RAS.

SHP2 not only mediates the RTK-stimulated activation of RAS [113] but also acts as a promoter of immune checkpoint pathways [114]. Certain SHP2 inhibitors are in early-phase clinical development for treating advanced or metastatic solid cancer (Table 1). TNO155 is an allosteric inhibitor that maintains SHP2 in a self-inhibited conformation [115]. Preclinical studies have shown promising anticancer activity from TNO155 combined with inhibitors of EGFR, MEK, ERK, CDK4/6, or KRAS-G12C and anti–PD-1 antibodies in xenograft models of NSCLC or CRC cells [116]. The efficacy and toxicity of combination regimens involving TNO155 together with TKIs or ICIs remain to be determined in clinical trials.

#### Inhibition of downstream signaling molecules

Oncogenic transformation mediated by RAS requires the downstream activation of the RAF/MEK/ERK and the PI3K/AKT/mTOR pathways. In theory, inhibition of any of these effectors should block oncogenic KRAS signaling. In fact, these two pathways intersect with each other and even form a feedforward loop to activate KRAS as an upstream signal [14]. Nonetheless, the success of targeting *KRAS*-mutant tumors by inhibiting single downstream molecules has been limited [18]. Despite these results, certain MAPK and PI3K pathway inhibitors have been approved or are entering clinical trials for combination therapies (Table 1) [88, 89]. In this section, we highlight some of these drugs and their application for *KRAS*-mutant cancers.

#### RAF inhibitors

The RAF family consists of ARAF, BRAF, and CRAF, all sharing RAS as a common upstream activator. Belvarafenib (HM95573) is a pan-RAF dimerization inhibitor that demonstrates selective anticancer activity with either cobimetinib or cetuximab in preclinical models, as well as in cancer patients with *RAS* or *RAF* mutations, especially melanoma patients (Table 1). *ARAF* mutations are conducive to resistance to bevacafenib, indicating that the RAF subtype has a compensatory function, and a secondary mutation of a RAF member may reactivate the MAPK pathway to avoid cell death [117].

LXH-254, an ATP-competitive inhibitor of BRAF and CRAF [118], is used in multiple clinical trials for patients with NSCLC or melanoma (Table 1). The anticancer activity of LXH-254 is demonstrated in tumors carrying *BRAF/RAS* co-mutations, but it has moderate activity against cancers driven by *KRAS* mutants [119]. ARAF may also mediate LXH-254 resistance in *RAS*-mutant cancer cell lines [119], supporting the hypothesis that all RAF isoforms need to be suppressed at the same time in order to achieve tangible antineoplastic effects.

Lifirafenib (BGB-283), a dual inhibitor of RAF and EGFR, is being used in clinical studies enrolling patients with *BRAF*- or *KRAS/NRAS*-mutated solid tumors [120]. Preclinical study suggests that lifirafenib enhances the antitumor activity of MEK inhibitors (mirdametinib and selumetinib) in *KRAS-*mutant tumors [121]. A phase I study on the safety and pharmacokinetics of the combination with lifirfenib and mirdametinib in *KRAS*-mutant NSCLC is ongoing (NCT03905148).

Other RAF inhibitors, such as PLX8394 and TAK-580 (MLN2480), are being evaluated as single-agent therapeutics in patients with advanced unresectable solid tumors (NCT02428712) or low-grade glioma (NCT03429803), respectively. Vemurafenib, dabrafenib, and encorafenib are RAF inhibitors approved for the treatment of tumors with *BRAF-V600E/K*, but not *RAS*, mutations. It appears that monotherapy with RAF inhibitors is not efficient against cancers with *KRAS* mutations, suggesting that combination with other MAPK pathway inhibitors should be attempted.

#### MEK inhibitors

Three MEK inhibitors, including trametinib (GSK1120212), cobimetinib (XL518), and binimetinib (MEK162), are approved in combination with BRAF inhibitors for the treatment of patients with advanced melanoma harboring *BRAF* mutations (*V600E* or *V600K*). Although the MEK inhibitor selumetinib (AZD6244) is not efficient against melanoma, it has recently been approved for the treatment of neurofibromatosis type 1 in children. Compared with standard treatments, MEK inhibitors alone are not efficient against solid tumors driven by *KRAS* mutations [122–125]. However, the combination of a MEK inhibitor and RAF inhibitor has shown promising activity against *KRAS-*mutant (especially *KRAS-G13D*) cells *in vitro* [126]. These findings provide the rationale for ongoing clinical trials that combine RAF (belvarafenib or LXH-254) and MEK inhibitors (cobimetinib or trametinib) to treat solid cancer with *KRAS* mutations (Table 1).

Two MEK inhibitors, pimisertib (MSC1936369B) and mirdametinib (PD0325901), are being evaluated for clinical activity against *KRAS*-mutant solid cancers (Table 1). Pimasertib alone is better than dacarbazine for improving progression-free survival in patient with *BRAF*- and *NRAS*-mutant melanoma [127, 128]. A combination of pimasertib and the MDM2 (a repressor of tumor suppressor TP53) inhibitor SAR405838 has shown preliminary antitumor activity in the treatment of solid cancers with *RAS* or *RAF* mutations [129]. Thus, targeting MEK combined with pharmacological TP53 induction may constitute a strategy for combating *KRAS*-mutant cancers.

#### ERK inhibitors

Although ERK inhibition is an effective strategy to overcome resistance to upstream MEK or RAF inhibitors [130–132], the clinical development of ERK inhibitors has been retarded when compared to that of MEK and RAF inhibitors. In 2020, the FDA granted an expanded access program for the ERK inhibitor ulixertinib (BVD-523) for the treatment of cancer patients with abnormal MAPK pathways, including but not limited to those involving *KRAS*, *NRAS*, *HRAS*, *BRAF*, *MEK*, and *ERK* mutations [133]. Ulixertinib is currently being evaluated in combination with hydroxychloroquine (an autophagy inhibitor) or palbociclib (a CDK4/6 inhibitor) in patients with advanced pancreatic and other solid tumors (NCT041452973 and NCT03454035).

Certain ERK inhibitors, such as LY3214996, GDC-0994, and MK-8353, alone or in combination with other drugs, are in the early stages of clinical development. LY3214996 is being used with abemaciclib, hydroxychloroquine, or RMC-4630, in solid tumors, including *KRAS*-mutant cancers (Table 1). Despite strong preclinical data [134], patients with advanced solid tumors cannot tolerate combination therapy with GDC-0994 and cobimetinib [135]. However, GDC-0994 alone has acceptable side effects and showed anticancer activity in two patients with *BRAF*-mutant CRC [136]. The toxicity of GDC-0994 in combination with other MAPK inhibitors needs to be further investigated.

MK-8353 shows a tolerable safety profile and antitumor activity in melanoma patients with a *BRAF-V600* mutation, rather than *RAS* mutation [137]. MK-8353 in combination with selumetinib or pembrolizumab is being investigated in patients with advanced malignancies, including CRC (Table 1). Although preclinical studies have shown that ERK mutations confer resistance to MAPK inhibitors [138], clinical studies have not yet reported the occurrence of acquired resistance to ERK inhibitors.

#### PI3K pathway inhibitors

Class I phosphatidylinositol-3-kinase (PI3K) is a lipid kinase that phosphorylates the signaling lipid phosphatidylinositol 4,5-bisphosphate (PIP2) to phosphatidylinositol-3,4,5-triphosphate (PIP3), resulting in the recruitment of protein kinase B (PKB, best known as AKT) to the plasma membrane and subsequent activation of mTOR for cell growth and proliferation [139]. In contrast, PTEN, a tumor suppressor, can convert PIP3 to PIP2, thereby diminishing PI3K activity. PI3K consists of a catalytic subunit (p110, including p110α, p110β, p110γ, and p110δ isoforms) and a regulatory subunit (p85α or p85β). Compared with p110γ and p110δ, which are mainly expressed in immune cells, the expression of p110α and p110β is common in various cells or tissues. PIK3CA, which encodes p110α, is frequently mutated in cancer as an important drug target [139]. *PIK3CA* mutations can coexist with *RAS* mutations, while *RAS* mutations and *MAPK* pathway mutations are usually mutually exclusive [140, 141]. These co-mutation patterns might guide clinical trials to target different signals from the MAPK and PI3K pathways.

In May 2019, the FDA approved the first drug, alpelisib, as a specific p110α inhibitor for treating breast cancer. The combination of alpelisib with other chemotherapy (capecitabine or paclitaxel) is being tested in patients with *PIK3CA*-mutant CRC or gastric cancer patients (Table 1). The secondary p110α inhibitor GDC-0077 is under clinical development for the treatment of breast cancer (NCT04632992). Additionally, copanlisib (a pan class I PI3K inhibitor), duvelisib (a p110γ/p110δ inhibitor), and idelalisib (a p110δ inhibitor) are approved to treat adult patients with relapsed or refractory follicular lymphoma, but not solid tumors, with *RAS* mutations. Despite considerable efforts to combine PI3K and MEK inhibitors in preclinical models [142], such a combination can cause significant toxicity in patients with *RAS*-mutated cancers [143, 144]. Similarly, the combination of AKT or mTOR inhibitors and MAPK inhibitors is generally poorly tolerated by patients, which may limit their applications [145, 146].

In summary, compared with MAPK pathway inhibitors, monotherapy or combination therapy with PI3K pathway inhibitors has limited benefits for patients with *KRAS*-mutated cancers, although such PI3K inhibitors may reverse resistance to KRAS-G12C inhibitors (discussed later). Current PI3K inhibitors are still challenged by insufficient selectivity, which results from the close structural resemblance among ATP binding sites of different PI3K isoforms [139].

#### Others

The MAPK and PI3K pathways activate transcription factors to induce or suppress the expression of genes involved in multiple cellular processes. Targeting related downstream processes (for example, autophagy, glycolysis, and immunosuppression) also may help to mitigate the carcinogenic activity of RAS but will not be discussed in this review. Of note, genomic screenings have enabled the discovery of synthetic lethal partners to inhibit tumor growth in *KRAS*-mutant cancer cells (Table 4).

**Table 4:** Genes involved in synthetic lethality of mutant KRAS-dependent cancers

Synthetic lethal genes	Full name	Main function	Tumor type	Reference
ANAPC1	Anaphase-promoting complex subunit 1	Mediates cell cycle progression	KRAS-mutant colon cancer	[147]
ARHGEF2	Rho/Rac guanine nucleotide exchange factor 2	Activates Rho-GTPases	KRAS-mutant pancreatic cancer	[148]
BCL2L1 (BCL-XL)	BCL2-like 1	Inhibits apoptosis	KRAS-mutant solid cancer	[149]
BIRC5 (survivin)	Baculoviral IAP repeat containing 5	Inhibits apoptosis	KRAS-mutant colon cancer	[150]
CDK1	Cyclin-dependent kinase 1	Mediates cell cycle progression	KRAS-mutant colon cancer	[151]
CDK4	Cyclin-dependent kinase 4	Mediates cell cycle progression	KRAS-mutant lung cancer	[152, 153]
DHODH	Dihydroorotate dehydrogenase (quinone)	Inhibits mitochondrial oxidative damage	KRAS-mutant pancreatic cancer	[154]
FGFR1	Fibroblast growth factor receptor 1	Mediates mitogenesis and differentiation	KRAS-mutant lung cancer	[155]
GATA2	GATA binding protein 2	Promotes development and survival	KRAS-mutant lung cancer	[156]
MAP3K7 (TAK1)	Mitogen-activated protein kinase kinase kinase 7	Promotes NF-κB activation	KRAS-mutant colon cancer	[157]
PLK1	Polo-like kinase 1	Promotes centrosome maturation and spindle assembly	KRAS-mutant chronic myelomonocytic leukemia or solid cancer	[147, 158, 159]
PRMT5	Protein arginine methyltransferase 5	Arginine methyltransferase	KRAS-mutant pancreatic cancer	[160]
PSMA5	Proteasome 20S subunit alpha 5	Mediates protein degradation	KRAS-mutant colon cancer	[147]
SHOC2	SHOC2 leucine-rich repeat scaffold protein	Promotes RAS signaling	KRAS-mutant leukemia and solid cancer	[161, 162]
SHP2 (PTPN11)	SH2 containing protein tyrosine phosphatase 2	Promotes RAS signaling	KRAS-mutant solid cancer	[113, 163]
SNAI2	Snail family transcriptional repressor 2	Promotes epithelial-mesenchymal transition	KRAS-mutant colon cancer	[164]
STK33	Serine/threonine kinase 33	Regulates cell cytoskeleton	KRAS-mutant solid cancer	[165]
TBK1	TANK binding kinase 1	Promotes NF-κB activation	KRAS-mutant lung cancer	[166]
WT1	WT1 transcription factor	Promotes development and survival	KRAS-mutant lung cancer	[167]
XPO1	Exportin 1	Mediates nuclear export	KRAS-mutant lung cancer	[168]
YAP1	Yes1-associated transcriptional regulator	Mediates the Hippo signaling pathway	KRAS-mutant pancreatic cancer	[167]

### Direct KRAS inhibition

#### Covalent KRAS-G12C inhibitors

Historically, KRAS was considered "undruggable" because it does not have a classical pocket suitable for binding small inhibitory molecules [18]. Recent structural studies and drug design efforts to produce G12Ci have changed this view (Table 5). The pioneering work of Shokat and colleagues uncovered a hidden pocket (switch-II) in the KRAS-GDP complex that is located next to the mutant cysteine 12 [172]. The proximity of switch-II to cysteine 12 facilitated the development of covalent inhibitors of switch-II, thereby achieving allosteric inhibition of cysteine 12 to prevent the nucleotide exchange catalyzed by GEF and diminish the subsequent interaction between RAS and RAF (Fig. 2c) [170, 172, 173]. Since wild-type KRAS lacks cysteine in the active site, the covalent inhibition of cysteine 12 is expected to be highly specific. ARS-1620 structurally modified from compound 12 [172], 1_AM [169], and ARS-853 [170] turned out to be the first G12Ci to elicit effective tumor suppression in patient-derived xenograft modes [171].

**Table 5:** Development history and application status of KRAS-G12C inhibitors

Name	Application date	Institutions	Structure	Status	Reference/ trial number
1_AM	August 2017	Dana-Farber Cancer Institute		Preclinical	[169]
Adagrasib	October 2019	Mirati		Clinical (approved)	[16] NCT04685135; NCT03785249; NCT04330664; NCT04793958
ARS-853	January 2016	Memorial Sloan Kettering Cancer Center		Preclinical	[170]
ARS-1620	January 2018	Wellspring Biosciences		Preclinical	[171]
Compound 12	November 2013	University of California		Preclinical	[172]
D-1553	October 2020	InventisBio	Structure not disclosed	Clinical (recruiting)	NCT04585035
GDC-6036	June 2020	Genentech	Structure not disclosed	Clinical (recruiting)	NCT04449874
JNJ-74699157	July 2019	Araxes/J&J	Structure not disclosed	Clinical (terminated)	NCT04006301
LY3499446	November 2019	Eli Lilly	Structure not disclosed	Clinical (terminated)	NCT04165031
Sotorasib	October 2019	Amgen		Clinical (approved)	[15] NCT04303780; NCT03600883; NCT04613596

**Fig. 5:**
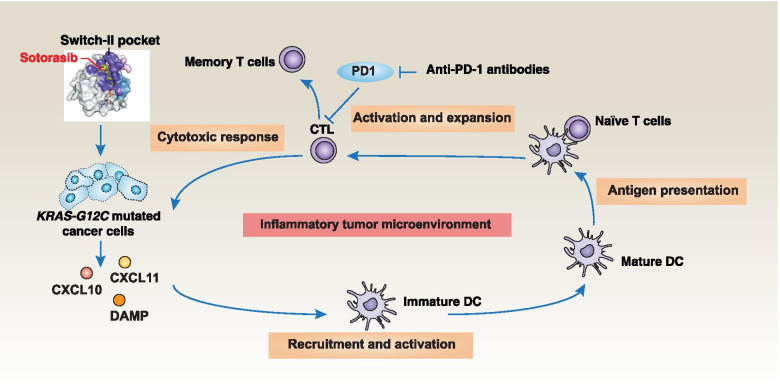
Immunostimulation by sotorasib acting on the tumor microenvironment. Sotorasib is a highly selective inhibitor of KRAS-G12C that reacts with mutant cysteine at position 12 by connecting to a structural feature called the switch II pocket. Sotorasib can induce the production of chemokines, such as C-X-C motif chemokine ligand 10 (CXCL10) and CXCL11, as well as the release of damage-associated molecular patterns (DAMPs), leading to dendritic cell (DC) maturation and activation. The priming of naive T cells to generate cytotoxic T lymphocytes (CTLs) requires mature DC-mediated antigen presentation. The number and function of tumor-targeted CTLs is a prerequisite for the immune system to attack cancer cells. However, the expression of immune checkpoint substances (such as programmed cell death protein 1 [PD-1]) limit the anticancer activity of CTLs, and the administration of anti–PD-1 antibodies reverses this process

Amgen and Mirati Therapeutics developed two structure-optimized covalent G12Ci formulations, sotorasib [15] and adagrasib [16]. Compared with ARS-1620, sotorasib and adagrasib have larger surface grooves, which enhance the effectiveness of irreversible interactions with the H95 residue in the 3 helix of KRAS-G12C protein [15, 16]. Sotorasib and adagrasib mediate selective tumor suppression activity across a panel of cancer cell lines harboring the *KRAS-G12C* mutation [14, 16, 174]. Durable responses to sotorasib have been observed in immunocompetent rather than immunodeficient tumor-bearing mice [175]. This may be explained by the fact that sotorasib induces the production of chemokines (CXCL10 and CXCL11) and potential damage-associated molecular patterns (DAMPs), leading to an immune response mediated by cytotoxic lymphocytes (Fig. 5) [175]. Accordingly, the combined use of anti–PD-1 antibodies further enhances sotorasib-induced tumor suppression in mouse models [175]. Whether G12Ci can be used to produce cancer-preventive or therapeutic immune responses is an open question. Interestingly, patient-derived xenograft models indicate that individual genetic alteration (such as in *KRAS*, *TP53, STK11,* or *CDKN2A)* cannot predict the anticancer activity of adagrasib [176]. CRISPR screens have identified negative (*MYC*, *SHP2*, *mTOR*, *RPS6*, *CDK1*, *CDK2*, *CDK4/6*, and *RB1*) and positive (*KEAP1* and *CBL*) regulators of adagrasib sensitivity in NSCLC cells [176]. Drug screening further revealed that a pan-EGFR family inhibitor (afatinib), a SHP2 inhibitor (RMC-4550), mTOR inhibitors (vistusertib and everolimus), and a CDK4/6 inhibitor (palbociclib) increase the response rate to adagrasib in cell cultures and mouse models [176], providing potential optimization strategies for translational research.

Clinical studies have shown some promising antitumor activity of sotorasib or adagrasib in patients with *KRAS-G12C-*mutated NSCLC that previously had been treated with platinum-based chemotherapy and/or PD-1/PD-L1 blockade. In a phase II trial, 37.1% (46/124) of such NSCLC patients responded to single-agent sotorasib (900 mg/kg, once daily) with a median duration of response of 11.1 months across all PD-L1 expression level subgroups (Table 6) [178]. The activity of sotorasib has been observed in patients with mutations in *TP53*, *STK11*, and *KEAP1* [178], which are associated with a poor prognosis in NSCLC [179]. Another phase I/II trial involved 129 *KRAS-G12C*–mutant cancer patients, in which 32.2% of NSCLC, 7.1% of CRC, and 14.3% of other tumor patients showed an objective response to sotorasib (Table 6) [177]. In May 2021, the FDA granted accelerated approval to sotorasib for the treatment of *KRAS-G12C*–mutated NSCLC. In June 2021, the FDA awarded breakthrough therapy designation to adagrasib for *KRAS-G12C*–mutated NSCLC based on an unpublished phase I/II study showing that 45% (23/51) of participants responded and 51% (26/51) of them were in stable conditions after using adagrasib (600 mg/kg, twice daily). Although the elimination half-lives of sotorasib (6 hours) and adagrasib (25 hours) are different, they have similar treatment-related adverse events (e.g., nausea, diarrhea, and vomiting). A number of clinical trials are underway to evaluate the antitumor activity of sotorasib or adagrasib alone or in combination with target drugs (docetaxel, pembrolizumab, cetuximab, afatinib, or TNO155) in solid cancers carrying *KRAS-G12C* mutations (Table 1). Specifically, two phase III trials will test the combination of sotorasib or adagrasib with docetaxel or cetuximab to treat *KRAS-G12C–*mutant NSCLC or CRC, respectively (NCT04685135 and NCT04793958).

**Table 6:** Clinical results of sotorasib therapy in advanced cancer with KRAS-G12C

	Hong et al., 2020 (n = 129) [177]	Skoulidis et al., 2021 (n = 126) [178]
**Characteristics**		
Median age (range, year)	62 (33–83)	63.5 (37–80)
NSCLC (n)	59	126
CRC (n)	42	0
Other solid cancer (n)	28	0
**Treatment**	Sotorasib (orally 180-960 mg/kg, once daily)	Sotorasib (orally 960 mg/kg, once daily)
**Efficacy**		
Objective response (%)	NSCLC: 32.2; CRC: 7.1; Other: 14.3	37.1
Disease control (%)	NSCLC: 88.1; CRC: 73.8; Other: 75.0	80.6
Complete response (%)	NSCLC: 0; CRC: 0; Other: 0	3.2
Partial response (%)	NSCLC: 32.2; CRC: 7.1; Other: 14.3	33.9
Stable disease (%)	NSCLC: 55.9; CRC: 66.7; Other: 60.7	43.5
Progressive disease (%)	NSCLC: 8.5; CRC: 23.8; Other: 14.3	16.1
Could not be evaluated (%)	NSCLC: 1.7; CRC: 2.4; Other: 7.1	1.6
**Adverse events**		
Any grade (%)	96.9	99.2
Serious (%)	45.0	45.3
Resulting in discontinuation of treatment (%)	7.0	7.1

The clinical development of other G12Ci compounds, such as GDC-6036 and D-1553, might provide additional opportunities for selectively targeting advanced solid tumors with *KRAS-G12C* mutations (Table 1). Notably, the clinical trial of two G12Ci formulations, LY3499446 and JNJ-74699157, has been terminated due to significant toxicity (NCT04165031 and NCT04006301). It remains to be seen whether these toxicities are caused by covalent or noncovalent off-targets.

#### Pan-KRAS inhibitors

BI-2852 is a pan-KRAS inhibitor that binds between the switch-I and switch-II pockets, thereby blocking the interaction of KRAS protein with GEF, GAP, and its downstream effectors [180]. Early preclinical studies confirmed its activity in blocking the KRAS pathway in NSCLC cells [180]. Using FR054 to inhibit glycosylation reactions further enhances the anticancer activity of BI-2852 against PDAC cells [181], supporting that the hexosamine biosynthesis pathway is a potential target for the treatment of *KRAS-*mutant cancers [182].

Revolution Medicines utilized a tri-complex technology platform to design a type of RAS(ON) inhibitor. RAS(ON) inhibitors (for example, RM-007 and RM-008) act as molecular glues to mediate protein-protein interactions between different mutant KRAS proteins and an endogenous protein (cyclophilin), thereby inhibiting the binding of mutant KRAS to SOS1 and effector proteins. Thus, the mode of action of RAS(ON) inhibitors is different from that of RAS(OFF) inhibitors, including G12Ci.

More recently, a small-molecule compound called Pen-cRaf-v1 has been identified as a pan-RAS inhibitor capable of targeting G12C and non-G12C RAS mutants to inhibit RAS-effector interaction [183]. Further animal studies are needed to determine the activity, metabolism, and toxicity of pan-KRAS inhibitors before their translational application into clinical medicine.

#### Others

An interesting trend in recent drug discovery is the selective induction of protein degradation through the proteasome. Proteolysis-targeting chimera (PROTAC) technology can be used to design new drugs that bridge the target protein to E3 ligases, hoping to achieve the target’s degradation to nonfunctional fragments. Whether this approach may be successful for the destruction of oncogenic RAS remains to be explored [184, 185].

### Mechanisms of adaptation or resistance to KRAS-G12C inhibitors

#### Producing new KRAS-G12C protein

**Fig. 6:**
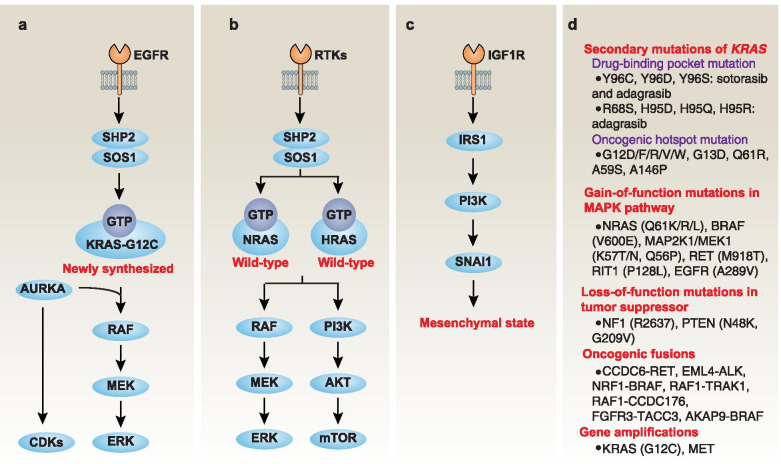
Mechanisms of adaptation or resistance to KRAS-G12C inhibitors. **a**. Production of new KRAS-G12C protein. Activation of the pathway involving epidermal growth factor receptor (EGFR)–SH2-containing protein tyrosine phosphatase 2 (SHP2)–SOS Ras/Rac guanine nucleotide exchange factor 1 (SOS1) is necessary to maintain the newly produced KRAS-G12C protein in an active GTP-bound form, which leads to the adaptation of ARS-1620 through the RAF-MEK-extracellular signal regulated kinase (ERK) pathway. The cell cycle regulator aurora kinase A (AURKA) can further enhance KRAS-G12C–mediated activation of mitogen-activated protein kinase (MAPK) effector pathways. **b.** Activating wild-type NRAS and HRAS. Multiple receptor tyrosine kinases (RTKs), rather than a single RTK, activate wild-type NRAS and HRAS, leading to acquired resistance to ARS-1620 and sotorasib by the RAF-MEK-ERK and the phosphatidylinositol 3-kinase (PI3K)-AKT-mechanistic target of rapamycin (mTOR) pathways. **c.** Inducing epithelial-to-mesenchymal transition (EMT). The insulin-like growth factor receptor (IGFR)-insulin receptor substrate 1 (IRS1) pathway mediates PI3K activation in a SHP2-independent manner, leading to acquired resistance to sotorasib or ARS-1620 through snail family transcriptional repressor 1 (SNAI1)-mediated EMT. **d.** Inducting secondary genetic alterations. An analysis of the genetic alterations of patients with acquired adagrasib resistance showed that 45% of the cases had a putative genetic mechanism of drug resistance. In short, acquired KRAS mutations in drug binding sites or oncogenic hotspots, gain-of-function mutations in the MAPK pathway, and loss-of-function mutations in tumor suppressor genes favor the acquisition of resistance to KRAS-G12C inhibitors

The activation of the EGFR-SHP2 pathway maintains newly synthesized KRAS-G12C protein in an active GTP-binding form, thereby leading to the adaptation of KRAS-G12C–mutated cancer cells to ARS-1620 (Fig. 6a) [186]. The cell cycle regulator aurora kinase A (AURKA) binds newly produced KRAS-G12C, which in turn stabilizes the interaction between CRAF and KRAS and mediates subsequent ERK effector signals for cell cycle progression [186]. Consequently, the inhibition of EGFR or AURKA reverses the adaptation of cancer cells to ARS-1620. These preclinical studies provide clues for the development of combined strategies that target EGFR or the cell cycle regulator to delay the development of resistance to KRAS-G12C inhibitors [186]. In addition to AURKA inhibitors (e.g., alisertib), many drugs that are already in clinical use or under development target various cell cycle regulators, especially CDKs. AURKA inhibitors and CDK inhibitors both have shown promise in the treatment of various types of cancer, including *KRAS*-mutant cancers [187–192].

#### Activating wild-type RAS

Wild-type and mutant RAS subtypes co-exist in the same cell, thus providing a feedback mechanism to reactivate RAS signaling if one of the two RAS pathways is blocked. As such, even if KRAS-G12C is effectively and completely inhibited, residual wild-type RAS (NRAS and HRAS) activity may confer resistance to G12Ci. Multiple RTKs (EGFR, HER2, FGFR, and c-MET), instead of a single RTK, activate wild-type RAS, resulting in acquired resistance to ARS-1620 and sotoracide (Fig. 6b) [193]. This feedback reactivation of wild-type RAS occurs in parallel to the neosynthesis of KRAS-G12C protein, resulting in drug resistance. Since SHP2 and SOS1 are the common nodes of RTK signals, SHP2 inhibitors (e.g., TNO155, SHP099, and RMC-4550) or SOS1 inhibitors (e.g., BAY-293) may either enhance the activity of G12Ci or reverse adaptive resistance. This hypothesis has been confirmed in preclinical models [83, 116, 186, 193–197], and such inhibitors are now entering clinical evaluation (NCT04330664).

#### Inducing epithelial-to-mesenchymal transition

Epithelial-to-mesenchymal transition (EMT), the process whereby epithelial cells are transformed into mesenchymal cells, is one of the acquired resistance mechanisms to antineoplastic therapies, including TKIs [198]. The induction of EMT in sotorasib-sensitive NSCLC cells by adding TGFβ or using transfection with SNAIL leads to acquired resistance to sotorasib through the activation of the PI3K pathway, which is not associated with significant AKT activation [199]. This suggests that the classical KRAS-PI3K-AKT pathway is not essential for acquired resistance to sotorasib, whereas KRAS-independent PI3K activation favors such resistance in lung cancer cells [200]. In the latter, the insulin-like growth factor receptor (IGFR)-IRS1 pathway, as a key upstream signal, mediates PI3K activation in a SHP2-independent manner, leading to acquired resistance to sotorasib or ARS-1620 in NSCLC cells (Fig. 6c) [199]. Therefore, the clinical optimization of G12Ci may profit from patient stratification based on EMT status.

In other cases, the activation of the PI3K-AKT-mTOR pathway clearly limits the efficacy of G12Ci, such as sotoracide or ARS1620, against NSCLC and PDAC cells [176, 201, 202]. Hence, the mechanisms of PI3K pathway-mediated resistance to G12Ci may depend on the tumor type and the degree of cellular (de)differentiation.

#### Inducing secondary genetic alterations

Specific secondary genetic alterations may provide additional information to predict G12Ci responses (Fig. 6d). Among 38 patients with *KRAS-G12C*-mutated solid cancers who received adagrasib monotherapy, 45% displayed a putative genetic mechanism for resistance [203]. The reactivation of RAS-MAPK signaling by 10 genetic alterations affecting the RAS-RAF-MEK-ERK pathway has been described in an NSCLC patient with acquired adagrasib resistance [204]. Secondary KRAS mutations, gain-of-function mutations of the MAPK pathway, loss-of-function mutations in tumor suppressor genes, gene fusion, and gene amplification are conducive to acquired resistance to G12Ci (Fig. 6d).

*In vitro* and *in vivo*, KRAS mutation studies further confirmed that the expression of clinically observed switch-II pocket mutations conferred resistance to adagrasib in *KRAS-G12C*–mutant BaF3 cells (a murine interleukin-3–dependent pro-B cell line) [203, 204]. Thus, the mutations *Y96C, Y96D,* or *Y96S* led to resistance to both adagrasib and sotorasib [203, 204]. In contrast, *H95D*, *H95Q,* and *H95R* mutants remained sensitive to sotorasib [203]. Interestingly, a RAS(ON) inhibitor retained its therapeutic index against cells harboring dual *G12C/Y96D* mutations [204], supporting the notion that RAS(ON) inhibitors mediate the inhibition of oncogenic RAS by a completely different mechanism.

Collectively, these studies provide proof of concept and mechanistic support for a combination therapy that suppresses adaptive genetic alterations. In fact, clinical trials evaluating adagrasib or sotorasib in combination with inhibitors of RTKs, MAPK, SOS1, or SHP2 are underway (NCT04330664 and NCT04185883) to explore novel strategies for overcoming cancer drug resistance [79, 205].

## Conclusions and future perspectives

With the development of G12Ci, we now have a tool to directly and irreversibly inhibit KRAS-G12C oncoprotein in patients [17]. However, the excitement spurred by this discovery has been dampened by the fact that the vast majority of patients fail to respond to G12Ci treatment due to primary or acquired resistance [177, 203, 204, 206]. Thus, there are still many outstanding problems to be solved.

### First, how can we develop next-generation inhibitors?

Different G12Ci compounds exhibit distinct activities and toxicities. For example, adagrasib was found to bind to KRAS-G12C equally in a series of different cell lines, despite the major variability in downstream signals [176]. The main risk of covalent inhibitors is the possibility of nonspecific reactions with off-target proteins containing cysteine residues [173]. Thus, the molecular identification of proteins accounting for off-target effects of G12Ci may improve structure-based drug design [203]. In addition to organ-mediated drug metabolism, tumor-resident microorganisms can directly decompose chemotherapeutic drugs to cause drug resistance [207]. Hence, optimizing the *in vivo* pharmacokinetic properties of KRAS inhibitors and evaluating the (in)activity of their metabolites is still an important area for examination. Finally, it remains to be seen whether it is possible to develop additional mutation-specific inhibitors or pan-mutant KRAS inhibitors [208].

### Second, how can we design combination therapies?

The design and implementation of strategies to minimize or overcome drug resistance is an important goal of clinical oncology in order to achieve complete and durable clinical responses [209]. The observed tumor heterogeneity and the extensive feedback between RAS and other tumor-related signals may promote drug resistance. The combination of several drugs intercepting different signaling pathways (e.g., upstream signaling, downstream signaling, parallel signaling, cell cycle processes, or immune checkpoints) may prevent or delay the development of therapy resistance, but usually at the cost of increased toxicity [14, 17]. A number of clinical trials combining G12Ci with other established agents (including TKIs and ICIs) are being launched. The design of such trials should avoid random combinations and follow a rationale based on the genetic, metabolic, and immune mechanisms of drug resistance.

### Third, how can we develop predictive cancer biomarkers?

Predictive biomarkers can guide treatment decisions by indicating the likely impact of a particular therapy on a patient. Some genetic alterations, especially secondary gene mutation, are associated with the development of adagrasib resistance in patients. In addition to using endoscopic ultrasound-guided fine needle aspiration, the analysis of circulating tumor DNA combined with next-generation sequencing technologies provides a way for examining the genetic characteristics of tumors. Despite these obvious technological advances, circulating metabolites or proteins should not be neglected as potential biomarkers, given that their quantitation would be much more convenient.

In summary, the development of ever more efficient and specific drugs blocking oncogenic RAS must be in parallel with major efforts to avoid and overcome therapeutic resistance. Given the continued efforts of industry, academia, and health care providers, as well as the latest breakthroughs in basic, clinical, and translational research on G12Ci, the prospects look bright.

## Data Availability

Not applicable.
